# Characterization of the first chloroplast genome of *Dichroa febrifuga* and its phylogenetic analysis

**DOI:** 10.1080/23802359.2021.1975506

**Published:** 2021-09-20

**Authors:** Wei Zhuo, Liqiang Wang, Xiaofu Zhu, Shenge Lu, Fengming Ren

**Affiliations:** a Research and Utilization on Characteristic Biological Resources of Sichuan and Chongqing Co-construction Lab, Chongqing Institute of Medicinal Plant Cultivation, Chongqing, China; b College of Pharmacy, Heze University, Heze, China

**Keywords:** Chloroplast genome, *Dichroa febrifuga*, medicinal plant, Hydrangeaceae, analysis

## Abstract

*Dichroa febrifuga*, seen as a medicinal plant, has a long history in traditional Chinese medicine. In this study, we adopted Illumina Hiseq sequencing technology in order to determine the first complete chloroplast (cp) genome of *D. febrifuga.* The cp genome was 157,647 bp in length, including a large single-copy (LSC) region of 86,728 bp, a small single-copy (SSC) region of 18,675 bp, and a pair of inverted repeat (IR) regions of 26,122 bp. The genome encoded 128 genes, including 84 protein-coding genes, 36 tRNA genes, and 8 rRNA genes. The phylogenetic analysis based on 20 complete cp genome sequences revealed that *D. febrifuga* was the sister of the ancestor of the reported Hydrangeeae species. The findings of the study will serve as a stepping stone for follow-up researches regarding the development of the *D. febrifuga* species.

*Dichroa febrifuga* Lour., a member of the genus *Dichroa* in Hydrangeaceae, grows in grove-shaded and humid mountainous areas, mainly distributing in Sichuan, Guizhou, Hunan, and Hubei of China. According to the ‘Pharmacopoeia of China’ (2020 version), *D. febrifuga* is one of the common Chinese herbal medicines in the folk, which can be used to treat malaria, and eliminate phlegm. At the same time, *D. febrifuga* is rich in quinazolone alkaloids, coumarins, steroids, polyphenols and other chemical components (Zhang et al. 2010). It not only has the effects of anti-malaria, anti-tumor, anti-insect (Han 2021), promoting wound healing and other pharmacological effects (Li et al. 2011), but is also a traditional Chinese medicine raw material used for developing antiparasitic drugs in veterinary medicine (Chen et al. 2021). However, as an important medicinal plant, there is no genomic information that has been reported so far. In this study, we reported and characterized the first complete chloroplast (cp) genome of *D. febrifuga* based on Illumina Hiseq sequencing.

Fresh leaves of *D. febrifuga* were collected from Nanchuan, chongqing, China (107°21′E, 29°13′N, 591 m). The voucher specimen was conserved in Chongqing Institute of Medicinal Plant Cultivation (accession number: CIMPC-RFM-20210303, Contact person: Fengming Ren; Email: 348080877@qq.com). In the experiment, the high-quality whole genomic DNA was extracted by the kit method (Beijing Kinco Biological Company). The purity and integrity of the extracted DNA were analyzed by Nanodrop (Thermo Fisher Scientific) and agarose gel electrophoresis. High quality DNA was used to generate paired-end libraries with insert size of 350 bp and about 2.94 Gb raw reads were generated by Illumina Hiseq 2500 Platform (Illumina, Hayward, CA, USA). The raw data from the platform was removed low-quality reads and adapters by trimmomatic (version 0.35) with default paprameters (Bolger et al. 2014). Using the clean data with 150 bp paired-end read length obtained from the raw data, a cp genome was assembled by NOVOPlasty (version 4.1) with the default parameters (Nicolas et al. 2016) and was annotated by CPGAVAS2 with default parameters (http://47.96.249.172:16019/analyzer/home) (Shi et al. 2019). After manual check and adjustment, the annotated cp genome was submitted to the GenBank (MW928534).

The complete *D. febrifuga* cp genomes was 157,647 bp in size and exhibited a typical angiosperm cp circular structure, containing four regions: large single-copy region (LSC: 86,728 bp), small single-copy region (SSC: 18,675 bp), and a pair of inverted repeat regions (IR: 26,122 bp). The GC contents of whole genome and each region of the genome were 37.86% (whole genome), 36.05% (LSC region), 31.68% (SSC region), and 43.09% (IR region). The GC content accorded with the typical characteristics of angiosperm cp genome. The cp genome encoded 128 functional genes, including 84 protein-coding genes, 36 tRNA genes, and 8 rRNA genes.

To reveal the phylogenetic relationship of *D. febrifuga* within Hydrangeeae, additional 19 species of Hydrangeeae from the NCBI website were selected to study. With *Ginkgo biloba* (NC_016986.1) and *Cycas revoluta* (NC_020319.1) as outgroups, a phylogenetic tree was built by using the 21 cp genome sequences. The 21 sequences were compared by MAFFT software (version 7.487) with the parameter of ‘–auto’ (Katoh and Standley 2013). Based on the aligned sequences, a Maximum-likelihood phylogenetic tree was built with 1000 bootstrap replicates by IQ-TREE (version 1.6.12) (Lam-Tung et al. 2015) under parameters of ‘-nt AUTO -m MFP -bb 1000 –bnni’. Phylogenetic analysis showed that *D. febrifuga* was the sister of the ancestor of the reported Hydrangeeae species. (Figure 1).

**Figure 1.**
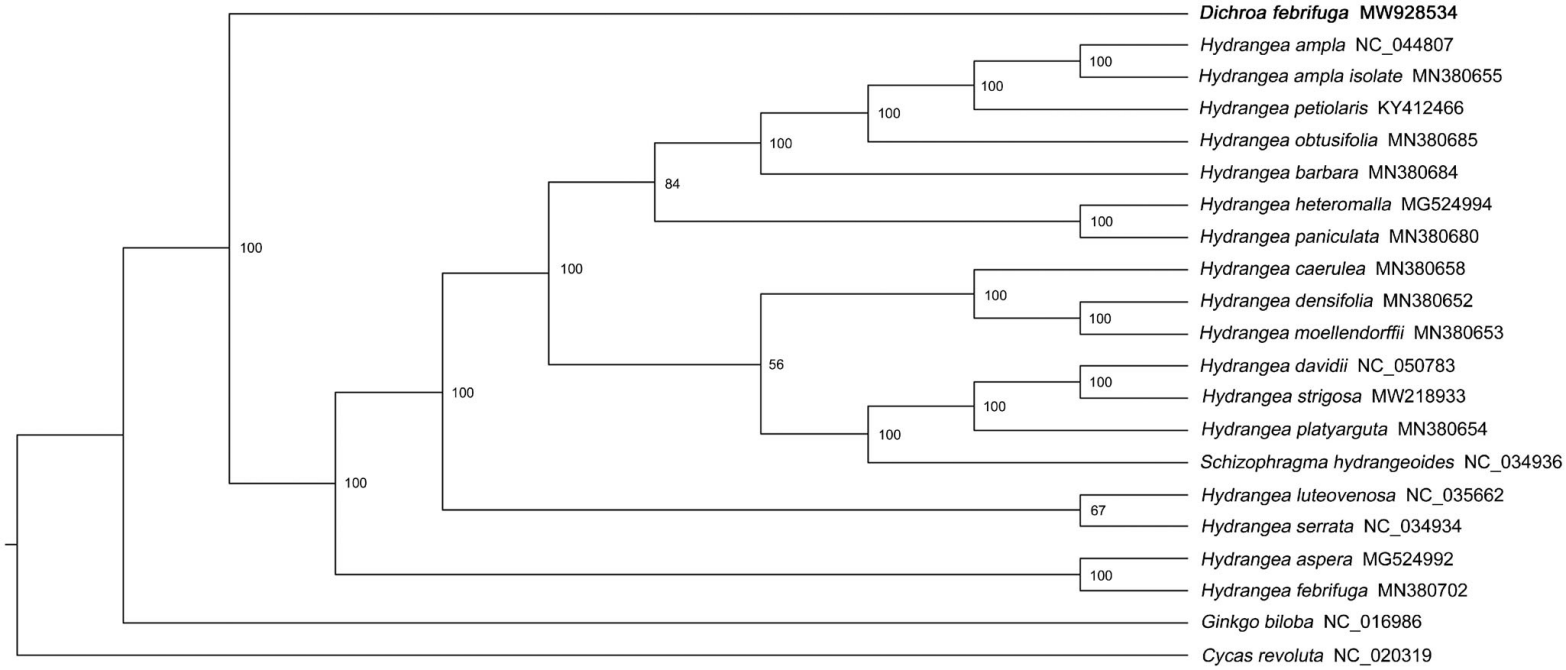
Maximum likelihood phylogenetic tree based on whole chloroplast genome sequences from 19 Hydrangeeae species with *Ginkgo biloba* (NC_016986) and *Cycas revoluta* (NC_020319) as outgroups. Bootstrap support values are shown above each branch. *D. febrifuga* is marked by bold font.

## Data Availability

The data that support the findings of this study are openly available in GenBank of NCBI at https://www.ncbi.nlm.nih.gov, under the accession number MW928534. The associated Bio-Project, Bio-Sample and SRA numbers are PRJNA543381, SAMN19416297 and SRR14688233, respectively.

## References

[CIT0001] Bolger AM, Marc L, Bjoern U. 2014. Trimmomatic: a flexible trimmer for Illumina sequence data. Bioinformatics. 30(15):2114–2120. 24695404 10.1093/bioinformatics/btu170 PMC4103590

[CIT0002] Chen DZ, Sun JH, Zhang JY, Zhou XZ. 2021. Discussion on the research and development direction of antiparasitic drugs for animals. J Trad Chinese Vet Med. 40(02):35–40.

[CIT0003] Han JH. 2021. Study on the prevention and treatment of broiler coccidiosis with Chinese herbal medicine *Dichroa febrifuga.* China Animal Health. 23(04):4–42.

[CIT0004] Katoh K, Standley DM. 2013. MAFFT multiple sequence alignment software version 7: improvements in performance and usability. Mol Biol Evol. 30(4):772–780. 23329690 10.1093/molbev/mst010 PMC3603318

[CIT0005] Lam-Tung N, Schmidt HA, Arndt VH, Bui QM. 2015. IQ-TREE: A fast and effective stochastic algorithm for estimating maximum-likelihood phylogenies. Mol Biol Evol. 32(1):268–274. 25371430 10.1093/molbev/msu300 PMC4271533

[CIT0006] Li Y, Liu MC, Jin LH, Hu DY, Sy Y. 2011. Research progress of *Dichroa febrifuga* chemical composition and biological activity. Guangzhou Chemical Industry. 39(09):7–9.

[CIT0007] Nicolas D, Patrick M, Guillaume S. 2016. NOVOPlasty: *de novo* assembly of organelle genomes from whole genome data. Nucleic Acids Res. 45(4):e18. 28204566 10.1093/nar/gkw955 PMC5389512

[CIT0008] Shi L, Chen H, Jiang M, Wang L, Wu X, Huang L, Liu C. 2019. CPGAVAS2, an integrated plastome sequence annotator and analyzer. Nucleic Acids Res. 47(W1):W65–73. 31066451 10.1093/nar/gkz345 PMC6602467

[CIT0009] Zhang Y, Li C, Lei GL. 2010. Study on the chemical constituents of *Dichroa febrifuga.* Chin J Exp Form. 16(05):40–42.

